# Supplementary material for: Probing the catalytic functions of Bub1 kinase using the small molecule inhibitors BAY-320 and BAY-524
Source: eLife. 2016 Feb 17;5:e12187. doi: 10.7554/eLife.12187 (PMC4769170; doi:10.7554/eLife.12187)
Supplement: Supplementary file 1. — IC50 profile of BAY-320 and BAY-524. DOI: http://dx.doi.org/10.7554/eLife.12187.018 [file elife-12187-supp1.docx]

|  | MW [g/mol] | Bub1 kinase inhibition  (2 mM ATP) | | HeLa proliferation | | HeLa proliferation (3 nM Paclitaxel ) | | A375 proliferation | A375 proliferation  (3 nM Paclitaxel ) |
| --- | --- | --- | --- | --- | --- | --- | --- | --- | --- |
|  |  | IC50 | SD | IC50 | SD | IC50 | SD | IC50 | IC50 |
| BAY-320 | 492.5 | 6.8E-07 | 2.8E-07 | 4.0E-06 | 1.4E-06 | 6.7E-07 | 2.7E-07 | 3.6E-06 | 4.0E-07 |
| BAY-524 | 482.5 | 4.5E-07 | 6.0E-08 | 8.1E-06 | 1.2E-06 | 6.4E-07 | 5.9E-07 | nd | nd |

concentrations are depicted in M
