# Supplementary material for: Probing the catalytic functions of Bub1 kinase using the small molecule inhibitors BAY-320 and BAY-524
Source: eLife. 2016 Feb 17;5:e12187. doi: 10.7554/eLife.12187 (PMC4769170; doi:10.7554/eLife.12187)
Supplement: Supplementary file 2. — In vitro kinase-selectivity profile of BAY-320 on a panel of 222 human kinases (Eurofins kinase profiler screen, Millipore). Shown are percentages of residual kinase activity at 10 µM BAY-320 and 10 µM ATP. DOI: http://dx.doi.org/10.7554/eLife.12187.019 [file elife-12187-supp2.docx]

| **Kinase** | **Activity*** |  | **Kinase** | **Activity*** |  | **Kinase** | **Activity*** |
| --- | --- | --- | --- | --- | --- | --- | --- |
| Abl(h) | 98 |  | Flt4(h) | 50 |  | PDK1(h) | 124 |
| ACK1(h) | 108 |  | Fms(h) | 76 |  | PhKγ2(h) | 38 |
| ALK(h) | 60 |  | Fyn(h) | 91 |  | Pim-1(h) | 56 |
| ALK4(h) | 82 |  | GCK(h) | 79 |  | Pim-2(h) | 119 |
| Arg(h) | 78 |  | GRK5(h) | 99 |  | Pim-3(h) | 112 |
| ARK5(h) | 85 |  | GRK6(h) | 102 |  | PKA(h) | 141 |
| ASK1(h) | 86 |  | GRK7(h) | 96 |  | PKBα(h) | 93 |
| Aurora-A(h) | 105 |  | GSK3α(h) | 148 |  | PKBβ(h) | 57 |
| Aurora-B(h) | 99 |  | GSK3β(h) | 209 |  | PKBγ(h) | 47 |
| Axl(h) | 91 |  | Haspin(h) | 47 |  | PKCα(h) | 102 |
| Blk(m) | 48 |  | Hck(h) | 110 |  | PKCβI(h) | 97 |
| Bmx(h) | 122 |  | HIPK1(h) | 105 |  | PKCβII(h) | 96 |
| BRK(h) | 72 |  | HIPK2(h) | 81 |  | PKCγ(h) | 100 |
| BrSK1(h) | 70 |  | HIPK3(h) | 102 |  | PKCδ(h) | 99 |
| BrSK2(h) | 108 |  | IGF-1R(h) | 50 |  | PKCε(h) | 106 |
| BTK(h) | 130 |  | IKKα(h) | 115 |  | PKCη(h) | 101 |
| CaMKI(h) | 41 |  | IKKβ(h) | 107 |  | PKCι(h) | 100 |
| CaMKIIβ(h) | 84 |  | IR(h) | 84 |  | PKCμ(h) | 107 |
| CaMKIIγ(h) | 106 |  | IRR(h) | 99 |  | PKCθ(h) | 88 |
| CaMKIδ(h) | 54 |  | IRAK1(h) | 105 |  | PKD2(h) | 90 |
| CaMKIIδ(h) | 89 |  | IRAK4(h) | 74 |  | PKG1α(h) | 89 |
| CaMKIV(h) | 77 |  | Itk(h) | 103 |  | PKG1β(h) | 82 |
| CDK1/cyclinB(h) | 68 |  | JAK2(h) | 206 |  | Plk1(h) | 127 |
| CDK2/cyclinE(h) | 76 |  | JAK3(h) | 146 |  | Plk3(h) | 112 |
| CDK3/cyclinE(h) | 92 |  | JNK1α1(h) | 89 |  | PRAK(h) | 102 |
| CDK5/p35(h) | 119 |  | JNK2α2(h) | 92 |  | PRK2(h) | 96 |
| CDK6/cyclinD3(h) | 80 |  | JNK3(h) | 46 |  | PrKX(h) | 65 |
| CDK7/cyclinH/MAT1(h) | 99 |  | KDR(h) | 82 |  | PTK5(h) | 224 |
| CDK9/cyclin T1(h) | 104 |  | Lck(h) | 32 |  | Pyk2(h) | 113 |
| CHK1(h) | 113 |  | LIMK1(h) | 95 |  | Ret(h) | 102 |
| CHK2(h) | 56 |  | LKB1(h) | 105 |  | RIPK2(h) | 85 |
| CK1γ1(h) | 114 |  | LOK(h) | 76 |  | ROCK-I(h) | 79 |
| CK1γ2(h) | 120 |  | Lyn(h) | 58 |  | ROCK-II(h) | 93 |
| CK1γ3(h) | 97 |  | MAPK1(h) | 94 |  | Ron(h) | 80 |
| CK1δ(h) | 104 |  | MAPK2(h) | 95 |  | Ros(h) | 92 |
| CK2(h) | 96 |  | MAPKAP-K2(h) | 77 |  | Rse(h) | 131 |
| CK2α2(h) | 110 |  | MAPKAP-K3(h) | 85 |  | Rsk1(h) | 72 |
| CLK2(h) | 115 |  | MEK1(h) | 96 |  | Rsk2(h) | 55 |
| CLK3(h) | 90 |  | MARK1(h) | 92 |  | Rsk3(h) | 99 |
| cKit(h) | 79 |  | MELK(h) | 58 |  | Rsk4(h) | 79 |
| CSK(h) | 76 |  | Mer(h) | 38 |  | SAPK2a(h) | 102 |
| c-RAF(h) | 78 |  | Met(h) | 62 |  | SAPK2b(h) | 103 |
| cSRC(h) | 111 |  | MINK(h) | 50 |  | SAPK3(h) | 105 |
| DAPK1(h) | 56 |  | MKK4(m) | 108 |  | SAPK4(h) | 106 |
| DAPK2(h) | 62 |  | MKK6(h) | 100 |  | SGK(h) | 63 |
| DCAMKL2(h) | 140 |  | MKK7β(h) | 136 |  | SGK2(h) | 64 |
| DDR2(h) | 81 |  | MLCK(h) | 50 |  | SGK3(h) | 46 |
| DMPK(h) | 110 |  | MLK1(h) | 82 |  | SIK(h) | 121 |
| DRAK1(h) | 50 |  | Mnk2(h) | 89 |  | Snk(h) | 101 |
| DYRK2(h) | 94 |  | MRCKα(h) | 117 |  | SRPK1(h) | 103 |
| eEF-2K(h) | 156 |  | MRCKβ(h) | 89 |  | SRPK2(h) | 102 |
| EGFR(h) | 75 |  | MSK1(h) | 47 |  | STK33(h) | 92 |
| EphA1(h) | 87 |  | MSK2(h) | 81 |  | Syk(h) | 64 |
| EphA2(h) | 114 |  | MSSK1(h) | 103 |  | TAK1(h) | 106 |
| EphA3(h) | 59 |  | MST1(h) | 84 |  | TAO1(h) | 74 |
| EphA4(h) | 88 |  | MST2(h) | 51 |  | TAO2(h) | 74 |
| EphA5(h) | 119 |  | MST3(h) | 33 |  | TAO3(h) | 80 |
| EphA7(h) | 89 |  | mTOR(h) | 100 |  | TBK1(h) | 102 |
| EphA8(h) | 116 |  | MuSK(h) | 55 |  | TGFBR1(h) | 98 |
| EphB2(h) | 106 |  | NEK2(h) | 97 |  | Tie2 (h) | 46 |
| EphB1(h) | 140 |  | NEK3(h) | 61 |  | TLK2(h) | 94 |
| EphB3(h) | 93 |  | NEK6(h) | 118 |  | TrkA(h) | 37 |
| EphB4(h) | 102 |  | NEK7(h) | 85 |  | TrkB(h) | 149 |
| ErbB4(h) | 128 |  | NEK11(h) | 83 |  | TSSK1(h) | 93 |
| FAK(h) | 89 |  | NLK(h) | 89 |  | TSSK2(h) | 95 |
| Fer(h) | 90 |  | p70S6K(h) | 50 |  | Txk(h) | 46 |
| Fes(h) | 91 |  | PAK2(h) | 89 |  | ULK2(h) | 94 |
| FGFR1(h) | 66 |  | PAK4(h) | 94 |  | ULK3(h) | 102 |
| FGFR2(h) | 102 |  | PAK5(h) | 120 |  | WNK2(h) | 84 |
| FGFR3(h) | 143 |  | PAK6(h) | 95 |  | WNK3(h) | 91 |
| FGFR4(h) | 134 |  | PAR-1Bα(h) | 103 |  | VRK2(h) | 104 |
| Fgr(h) | 143 |  | PASK(h) | 44 |  | Yes(h) | 100 |
| Flt1(h) | 40 |  | PDGFRα(h) | 102 |  | ZAP-70(h) | 120 |
| Flt3(h) | 97 |  | PDGFRβ(h) | 99 |  | ZIPK(h) | 56 |

* % residual kinase activity at 10 µM BAY-320
